# Orcinol Glucoside Improves Senile Osteoporosis through Attenuating Oxidative Stress and Autophagy of Osteoclast via Activating Nrf2/Keap1 and mTOR Signaling Pathway

**DOI:** 10.1155/2022/5410377

**Published:** 2022-05-09

**Authors:** Wan Gong, Mengqin Liu, Qi Zhang, Quanlong Zhang, Yang Wang, Qiming Zhao, Lu Xiang, Chengjian Zheng, Qiaoyan Zhang, Luping Qin

**Affiliations:** ^1^School of Pharmaceutical Sciences, Zhejiang Chinese Medical University, Hangzhou 310053, China; ^2^School of Basic Medical Sciences, Zhejiang Chinese Medical University, Hangzhou 310053, China; ^3^Zhejiang Traditional Chinese Medicine & Health Industry Group Co., Ltd., Hangzhou 310016, China; ^4^School of Life Sciences, Zhejiang Chinese Medical University, Hangzhou 310053, China; ^5^School of Pharmacy, Second Military Medical University, Shanghai 200433, China

## Abstract

Oxidative stress and autophagy play essential roles in the development of senile osteoporosis which is characterized by disrupted osteoclastic bone resorption and osteoblastic bone formation. Orcinol glucoside (OG), a phenolic glycoside isolated from *Curculigo orchioides* Gaertn, possesses antiosteoporotic properties. This study examined the protective effects of OG on bone loss in SAMP6 mice and explored the underlying mechanisms. The osteoporotic SAMP6 mice were treated with OG oral administration. RAW264.7 cells were induced to differentiate into osteoclast by RANKL and H_2_O_2_ in vitro and received OG treatment. The results demonstrated that OG attenuated bone loss in SAMP6 mice and inhibited the formation and bone resorption activities of osteoclast and reduced levels of oxidative stress in bone tissue of SAMP6 mice and osteoclast. Furthermore, OG activated Nrf2/Keap1 signaling pathway and enhanced the phosphorylation of mTOR and p70S6K which are consequently suppressing autophagy. Of note, the effect of OG on Nrf2/Keap1 signaling was neutralized by the mTOR inhibitor rapamycin. Meanwhile, the inhibitory effect of OG on autophagy was reversed by the Nrf2 inhibitor ML385.Conclusively, OG attenuated bone loss by inhibiting formation, differentiation, and bone resorption activities of osteoclast. Regulation of Nrf2/Keap1 and mTOR signals is a possible mechanism by which OG suppressed oxidative and autophagy of osteoclasts. Thus, OG prevented senile osteoporosis through attenuating oxidative stress and autophagy of osteoclast via activating Nrf2/Keap1 and mTOR signaling pathway.

## 1. Introduction

Senile osteoporosis (SOP), which is the most common age-related skeletal disorder, is characterized by low bone mass and microstructural destruction, leading to an increase in bone fragility and propensity to fracture [1]. SOP, which belongs to the primary osteoporosis, usually occurs in a population over 65 years old and results from the chronic and progressive degradation of the functional capacities of cells and tissue-related with bone metabolism [2]. Oxidative stress has been a known inducer of senescence and has been linked to the incidence of osteoporosis [3]. Increased oxidative stress disturbs the coordinated action of osteoclastic bone resorption and osteoblastic bone formation, leading to bone loss [4]. Antioxidants prevent senescence and contribute to activating the differentiation and mineralization of osteoblasts and reducing the osteoclast activity and then maintaining the balance of bone remodeling [5]. Therefore, antioxidant treatment could be a potential strategy for the therapy of senile osteoporosis.

Osteoclast, a multinucleated cell derived from the monocyte/macrophage lineage [6], is the only discovered cell with the capacity of bone resorption [7]. Oxidative stress activates the differentiation of preosteoclasts and strengthens the bone resorption as demonstrated by increases in the activity of tartrate-resistant acid phosphatase (TRAP) in osteoclasts and the bone resorption area of osteo-surface well [8]. Autophagy is a catabolic process, through which damaged organelles and some cellular molecules including protein aggregates are removed by lysosomal digestion [9]. Increasing evidence displays that autophagy dysregulation results in alteration of osteoclast function and increased bone loss [10]. Under oxidative stress, autophagy is activated [11] and accompanied by an unusual increase in osteoclast differentiation and bone resorption [12]. Thus, the interplay between oxidative stress and autophagy plays a crucial role in cellular homeostasis and the survival of osteoclast. Therefore, inhibition of autophagy may retard the activation of osteoclasts resulting from excess reactive oxygen species (ROS).

Under oxidative stress, the nuclear factor erythroid 2-related factor 2 (Nrf2)/kelch-like ECH-associated protein 1 (Keapl) pathway is activated to modulate the gene expression of encoding antioxidant and detoxifying enzymes to maintain cellular redox homeostasis [13]. Meanwhile, autophagy is regulated by the mammalian target of rapamycin (mTOR), and the function of mTOR is to inhibit autophagy [14]. In response to oxidative stress, Nrf2 is involved in the fine-tuning of the autophagic process [15]. In fact, mTOR is a key regulator of osteoclast function, mTOR activation in osteoclast prevents bone loss in a mouse model of osteoporosis [16]. Nrf2 signaling is involved in the regulation of osteoclast formation and activity. Overexpression of Nrf2 helps to increase the activities of antioxidant enzymes induced by the receptor activator of nuclear factor-*κ* B ligand (RANKL) and inhibit osteoclast differentiation [17]. Hence, Nrf2/mTOR, as well as their interconnection, is of great importance to bone homeostasis and might modulate osteoclast function under oxidative stress.

In recent years, traditional herbs are attracting increasing attention due to their antioxidation and regulatory effects on autophagy. Orcinol glucoside (OG) is a natural phenolic glycoside from *Curculigo orchioides* Gaertn. which has long been used for the treatment of osteoporosis and postmenopausal syndrome in China [18]. OG has been reported as an active nature compound with several pharmacological effects, including antioxidant, anxiolytic, and antidepressive activities [19, 20]. A previous study demonstrated that OG could enhance MSC differentiation into osteoblasts and prevent adipogenesis via Wnt/*β*-catenin signaling pathway [18]. Our previous study found that OG decreased bone loss in ovariectomized rats and inhibited the formation and differentiation of osteoclast [21]. However, the effects of OG on senile osteoporosis and the underlying mechanism of inhibiting osteoclast differentiation remain obscure.

In the present study, we investigated the antiosteoporotic effects and potential mechanism of OG in the senescence-accelerated mice-prone 6 (SAMP6) and osteoclast derived from RAW264.7 under oxidative stress and found that OG improved BMD and bone microarchitecture, mitigated oxidative stress in SAMP6 mice, and suppressed functions of osteoclast by attenuating oxidative stress and autophagic flux via Nrf2 and mTOR pathway. Our current study suggests that OG may be a therapeutic option for ameliorating senile osteoporosis.

## 2. Materials and Methods

### 2.1. Reagents

Orcinol glucoside was purchased from Ding Rui Chemical Co., Ltd. (Shanghai, China). Rapamycin (RAP), ML385, and N-acetyl-L-cysteine (NAC) were purchased from Shanghai Yuanye Bio-Technology Co., Ltd. (Shanghai, China). Biochemistry kits for TRAP, carboxy-terminal cross-linking telopeptide of type 1 collagen (CTX-1), cathepsin K (CTSK), calcium (Ca^2+^), deoxypyridine (DPD), ROS, *γ*-glutamylcysteine synthetase (GCS), heme oxygenase-1(HO-1), NADPH oxidase 1,2,4 (NOX1, NOX2, NOX4), NAD(P)H: quinone reductase (NQO-1), and glucose-6-phosphate dehydrogenase (G6PDH) were purchased from Nanjing Jiancheng Bioengineering Institute (Nanjing, Jiangsu, China). A Cell Counting kit-8 (CCK8) kit, 4′,6-diamidino-2-phenylindole (DAPI), BCA protein assay kit, and cell lysis buffer for Western Blotting were purchased from Beyotime Biotechnology (Shanghai, China). A TRAP staining kit and rhodamine-conjugated phalloidin were purchased from Sigma-Aldrich (St. Louis, MO, USA). Dulbecco's modified eagle medium (DMEM), *α*-modified minimal essential medium(*α*-MEM), fetal bovine serum, and penicillin/streptomycin were purchased from Gibco (Invitrogen Corporation). RANKL was purchased from Pepro Tech (Rocky Hill, NJ, USA). Antibodies against c-FOS (#BA0207-2) and MMP-9 (#BA2202) were purchased from Boster Biological Technology (Wuhan, China). Antibodies against NOX1 (#ab131088), NOX2 (#ab129068), NOX4 (#ab133303), CTSK (#ab19027), and SOD2 (#ab13533) were purchased from Abcam (Cambridge, MA, USA). Antibodies against NFATc1 (#8032S), Nrf2 (#12721S), Keap1 (#8047S), SQSTM1/p62 (#23214S), LC3 (#4108S), Beclin1 (#3495S), ATG5 (#12994T), ATG7 (#8558 T), ATG12 (#4180 T), p-mTOR (#5536S), and mTOR (#2983S) for Western blot were purchased from Cell Signaling Technology (Danvers, MA, USA). Antibodies against p-p70S6K (#AF3228), p70S6K (#AF6226), GAPDH (AF7021), and anti-rabbit IgG HRP linked antibody (#S0001) were purchased from Affinity Biosciences Co., Ltd. (Cincinnati, OH, USA). Ad-mRFP-GFP-LC3 was obtained from Hanbio Biotechnology Co., Ltd. (Shanghai, China).

### 2.2. Animals and Treatment

The male SAMP6 and senescence-accelerated mice-resistant 1 (SAMR1) strain at 4 months of age were obtained from the Department of Experimental Animal Science in Peking University Health Science Center (Beijing, China) and maintained at the Experimental Animal Center of Zhejiang Chinese Medical University (Hangzhou, China). The mice were kept with good ventilation and a 12 h light/dark cycle and were retained with feeding and drinking freely before being sacrificed.

After one week of acclimatization, SAMR1 mice were used in the control group (CON), SAMP6 mice were randomized into four groups including model group (MOD), positive control group (NAC,100 mg/kg NAC, i.g. per day), low-dose OG group (OG-50, 50 mg/kg OG, i.g. per day), and high-dose OG group (OG-100, 100 mg/kg OG, i.g. per day). The mice in the control group and model group were orally administered with 0.5% CMC-Na, and others were orally given NAC or OG according to the experimental protocols. After a 10-week treatment, urine and blood samples were collected for biochemical assays. Then, all mice were euthanized to obtain tissues. The femurs of the right hind were fixed in 4% paraformaldehyde (PA) for microcomputed tomography (micro-CT) scanning. The bones of the forelimb were collected for Western blot analysis.

### 2.3. Micro-CT and Histological Assessments

The right distal femurs were fixed in 4% PA and measured with a micro-CT system (eXplore Locus, GE Healthcare, USA). Micro-CT was carried out for the assessment of the femur microstructure in terms of bone mineral density (BMD), bone surface to bone volume (BS/BV), bone surface to tissue volume (BS/TV), bone volume to tissue volume (BV/TV), trabecular thickness (Tb. Th), trabecular separation (Tb. Sp), trabecular number (Tb. N), structure model index (SMI), and connectivity density. The Explore Reconstruction Utility software (GE Healthcare, USA) was employed in 3D reconstruction and data processing.

Fixed femurs were decalcified in 10% EDTA for 1 month and embedded into paraffin blocks. Then, the femurs were cut into 5 *μ*m thick histological sections and used for TRAP staining. The slices were photographed using a microscope (DM4B, Leica, Wetzlar, Germany).

### 2.4. Measurement of Serum and Urine Biomarkers

The serum levels of TRAP, CTX-1, CTSK, and urine DPD were measured following instructions of the ELISA kits. Concentrations of urine Ca^2+^ were measured by colorimetry with a commercial kit.

### 2.5. Cell Culture

The murine macrophage RAW264.7 cell line was purchased from the Cell Bank of the Chinese Academy of Sciences (Shanghai, China). Cells were cultured in DMEM medium containing 10% fetal bovine serum and 1% (*v*/*v*) penicillin and streptomycin at 37°C in humidified air containing 5% CO_2_. For osteoclast differentiation, RAW264.7 cells were incubated in complete *α*-MEM media containing 25 ng/mL RANKL for 36 h, then incubated with 20 *μ*M H_2_O_2_ for another 36 h. Osteoclasts were identified with a TRAP kit.

### 2.6. Cell Viability Assay

Cell viability was evaluated with the CCK-8 assay. Briefly, RAW264.7 cells were seeded in 96-well plates (2 × 10^4^ cells/well), then treated with OG in different concentrations or NAC for 72 h with or without 20 *μ*M H_2_O_2_. In following treatment, fresh medium with 10% CCK-8 solution was added to the cells, which were then incubated at 37°C for 30 min. Absorbance was immediately detected at 450 nm with a spectrophotometer (Thermo Fisher Scientific, Shanghai, China).

### 2.7. TRAP Staining and Activity Assay

RAW 264.7 cells were seeded in 96-well plates (1 × 10^4^ cells/well) and incubated with OG in different concentrations or NAC in the process of differentiation. Thereafter, cells were fixed with 4% PA for 30 min and stained with a TRAP kit. TRAP-positive cells (with more than three nuclei) were counted as osteoclasts. TRAP activities were measured using the p-nitrophenyl sodium phosphate method [22].

### 2.8. F-Actin Ring Formation Assay

RAW264.7 cells were seeded in dishes (6 × 10^5^ cells/dish), incubated with RANKL and different concentrations of OG or NAC for 24 h, and further treated with H_2_O_2_ for 12 h. Then, cells were fixed with 4% PA for 30 min, labeled with 5 *μ*g/mL rhodamine-conjugated phalloidin at 37°C for 40 min. Cell nuclei were stained with DAPI for 10 min prior to visualizing F-actin ring of osteoclasts under a confocal fluorescence microscope (LSM880, Carl Zeiss, Oberkochen, Germany).

### 2.9. Osteoclastic Bone Resorption Assay

RAW264.7 cells were seeded on bovine bone slices (obtained from bovine cortical femur bone: 5.5 mm diameter and 0.4 mm thickness) in 96-well plates. After adhered, cells were treated with RANKL and OG or NAC for 36 h and further incubated with H_2_O_2_ for 3 days. The levels of Ca^2+^ and CTX-1 in cell culture media were measured with assay kits.

### 2.10. Intracellular ROS Detection

RAW264.7 cells were seeded in 96-well plates for intracellular ROS determination. Cells were treated with RANKL and OG in different concentrations or NAC for 36 h and then with H_2_O_2_ for another 24 h. Subsequently, cells were labeled with 10 *μ*M 2′,7′-dichlorofluorescein diacetate (DCFH-DA) at 37°C for 20 min. The fluorescence intensity of 2′,7′-dichlorofluorescein produced from the oxidation of DCFH-DA stimulated by intracellular ROS was immediately detected with a Synergy HT spectrophotometer (Bio Tek, Winooski, VT, USA).

### 2.11. Enzyme-Linked Immunosorbent Assay

RAW264.7 cells were seeded in 48-well plates (2 × 10^4^ cells/well) and then treated with RANKL and OG or NAC for 36 h and then with H_2_O_2_ for 24 h. Afterward, cells were lysed on ice for 30 min and centrifuged for 20 min at 4°C (12000 rpm/min). Then, the supernatant was collected for measuring the activities of NOX1, NOX2, NOX4, GCS, NQO-1, HO-1, and G6PD with ELISA kits following the manufacturer's guides.

### 2.12. Protein Extraction and Western Blot Analysis

RAW 264.7 cells were seeded in 6-well plates (2 × 10^6^ cells/well). Cells were treated with RANKL and different concentrations of OG or NAC or coincubation of OG with 5 *μ*M RAP or ML385 for 48 h and then with H_2_O_2_ for another 4 h.

Total proteins in cultured cells or forelimb bones of mice were extracted using lysis buffer containing proteases and phosphatase inhibitors. Western blot was performed as previously described [23] by using the following antibodies: anti-NFATc1, anti-c-FOS, anti-CTSK, anti-MMP9, anti-NOX1, anti-NOX2, anti-NOX4, anti-SOD2, anti-Nrf2, anti-Keap1, anti-p62, anti-LC3, anti-Beclin1, anti-ATG5, anti-ATG7, anti-ATG12, anti-p-mTOR, anti-mTOR, anti-p-p70S6K, anti-p70S6K, and anti-GAPDH. Blots were presented by E-Gel Imager (Tanon-5200 Multi, Shanghai, China). The intensity of band was measured with Image J software.

### 2.13. Immunofluorescence Analysis

The nucleus translocation of Nrf2 was evaluated with immunofluorescence analysis. The detailed protocol was described previously [17]. After incubation with the secondary antibody, images were acquired by a confocal fluorescence microscope.

### 2.14. Analysis of Autophagic Flux

The autophagic flux was determined via detecting LC3 puncta using a confocal microscope. RAW264.7 cells were seeded in dishes and transiently transfected with recombinant adenovirus mRFP-GFP-LC3 according to the manufacturer's instructions. Then, cells were treated with RANKL, H_2_O_2_, and OG as described above. The cells were pretreated with rapamycin for 1 hour before OG exposure. Puncta were detected by a laser scanning confocal microscope. Autophagy flux was quantitated according to the number of GFP, mRFP, and the merged dots per cell, and 20 cells were calculated in each group.

### 2.15. Statistical Analysis

Results were presented as the mean ± standard deviation (SD). The statistical analysis was performed with the GraphPad Prism 8.0.2 software (GraphPad Software, CA) using *t*-test or one-way analysis of variance (ANOVA). Differences between treatments were considered to be statistically significant at *P* < 0.05.

## 3. Results

### 3.1. OG Attenuates Bone Loss in SAMP6 Mice

**Figure 1:**
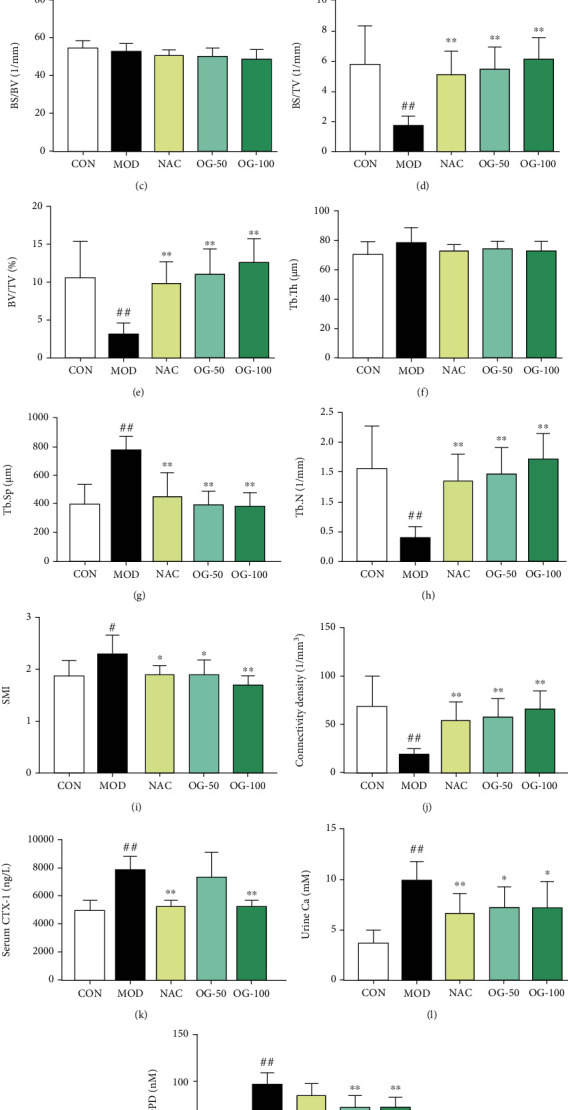
Orcinol glucoside (OG) attenuates bone loss in SAMP6 mice. (a) Representative 3D micro-CT images of the distal femoral trabecular bone microarchitecture; (b) bone mineral density (BMD); (c) bone surface to bone volume (BS/BV); (d) bone surface to tissue volume (BS/TV); (e) bone volume to tissue volume (BV/TV); (f) trabecular thickness (Tb. Th); (g) trabecular separation (Tb. Sp); (h) trabecular number (Tb. N); (i) structure model index (SMI); (j) connectivity density; (k) serum CTX-1 levels were determined by ELISA; (l, m) urine Ca^2+^ and DPD levels were measured by biochemical kits. CON: control group, SAMR1 mice treated with 0.5%CMC-Na; MOD: model group, SAMP6 mice treated with 0.5%CMC-Na; NAC: SAMP6 mice treated with NAC (100 mg/kg); OG-50: SAMP6 mice treated with OG (50 mg/kg); OG-100: SAMP6 mice treated with OG (100 mg/kg). The data are expressed as mean ± SD (*n* = 8‐9). ^#^*P* < 0.05, ^##^*P* < 0.01 vs. control group. ^∗^*P* < 0.05, ^∗∗^*P* < 0.01 vs. model group.

The hind femur on the right side was used for micro-CT analysis. The reconstructed 3D images exhibited a distinct bone loss in SAMP6 mice and the treatment with OG inhibited bone loss (Figure 1(a)). In the quantitative analysis, BMD was found to be prominently increased in the OG treatment group (Figure 1(b)). The results showed that BS/TV, BV/TV, and Tb. N were obviously increased while Tb. Sp was decreased in the OG treatment group compared with the model group (Figures 1(d), 1(e), and 1(h)). However, BS/BV and Tb. Th displayed no statistical difference between the model and OG treatment groups (Figures 1(c) and 1(f)). Meanwhile, as explicitly shown in Figures 1(i) and 1(j), OG markedly reduced the values of SMI and increased connectivity density in SAMP6 mice. In addition, the levels of serum CTX-1 (Figure 1(k)), urine Ca^2+^ (Figure 1(l)), and DPD (Figure 1(m)) in the model group were significantly increased compared with the control group (*P* < 0.01), which were suppressed by OG and NAC treatment. Collectively, these results suggested that OG was capable of preventing bone loss in SAMP6 mice.

### 3.2. OG Inhibits Activity and Differentiation of Osteoclast in SAMP6 Mice and RAW 264.7 Cells

**Figure 2:**
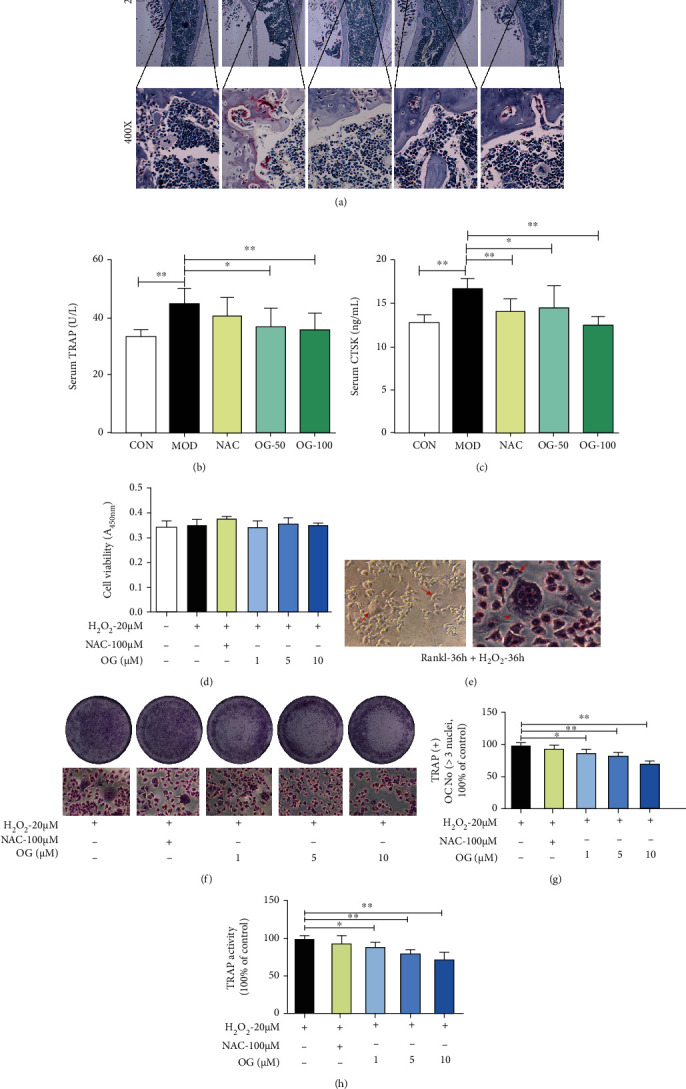
Orcinol glucoside (OG) inhibits activity and differentiation of osteoclast in SAMP6 mice and RAW264.7 cells. (a) TRAP staining images of the tibias. (b, c) Serum TRAP and CTSK activity were determined by ELISA (*n* = 8‐9). (d) The effect of OG on cell viability was detected by CCK-8 kits. (e) The morphological feature of osteoclast derived from RAW264.7 cells under a microscope (400x). (f) Representative TRAP staining images of osteoclast for treated with RANKL and OG and subsequently treated with H_2_O_2_. (g) The number of TRAP-positive multinucleated (>3 nuclei) osteoclasts. (h) TRAP activities in osteoclast. Images presented are representative of ≥3 sections for each group or independent experiments, and data are expressed as mean ± SD. ^∗^*P* < 0.05, ^∗∗^*P* < 0.01 vs. the indicated group.

TRAP staining was used to observe histological changes. TRAP-stained sections showed a marked increase in osteoclasts on the bone surface of SAMP6 mice. Treatment with OG inhibited bone loss by decreasing the number of TRAP-positive osteoclasts on the bone surface in a dose-dependent manner (Figure 2(a)). The levels of serum TRAP (Figure 2(b)) and CTSK (Figure 2(c)) in the model group were significantly increased compared with the control group (*P* < 0.01), which were suppressed by OG and NAC treatment.

To explore the effect of OG on the proliferative activity of preosteoclasts in vitro, CCK-8 assay was performed. As a result, we found that the treatment with 1, 5, and 10 *μ*M OG for 72 h and 20 *μ*M H_2_O_2_ exerted no cytotoxic effects on RAW264.7 cells (Figure 2(d)). Then, RAW264.7 cells were stimulated with 25 ng/mL RANKL and 20 *μ*M H_2_O_2_ to differentiate into osteoclasts. As indicated in Figure 2(e), apparent multinuclei and pseudopodia were displayed in cells, demonstrating that these cells were typical osteoclasts. In addition, quantitative data showed that OG decreased the number of osteoclasts and the activity of TRAP in a dose-dependent manner (Figures 2(g) and 2(h)). Altogether, these results indicate that OG could potentially inhibit osteoclastogenesis by affecting osteoclast formation and differentiation.

### 3.3. OG Suppresses Bone Resorption in SAMP6 Mice and Osteoclast Derived from RAW264.7 Cells

**Figure 3:**
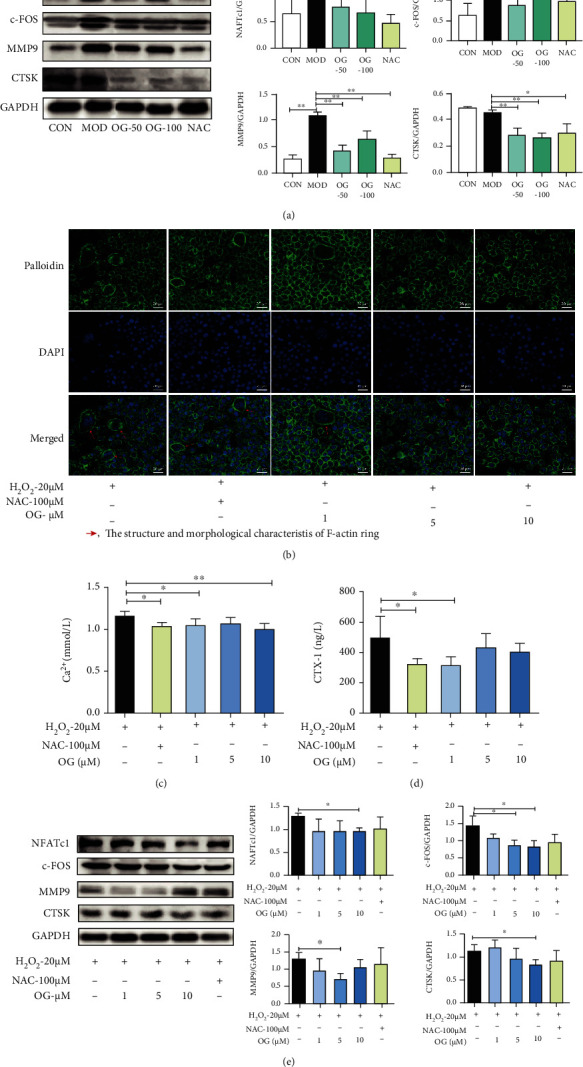
OG suppresses bone resorption in SAMP6 mice and osteoclast derived from RAW264.7 cells. (a) Representative Western blot analysis and the quantification of NFATc1, c-FOS, CTSK, and MMP9 expression in bone tissue of SAMP6 mice. (b) Osteoclasts were labeled with phalloidin (F-actin ring) and DAPI (nuclei). Scale bars: 20 *μ*m. (c, d) The content of Ca^2+^ and CTX-1 in the coculture medium of osteoclast and bone slices. (e) Representative Western blot analysis and the quantification of NFATc1, c-FOS, CTSK, and MMP9 expression in osteoclast. Images presented are representative of ≥3 sections for each group or independent experiments, and each point represents mean ± SD. ^∗^*P* < 0.05, ^∗∗^*P* < 0.01 vs. the indicated group.

The effect of OG on the protein level of the osteoclast markers was examined by Western blotting. OG treatment obviously suppressed the expression of NFATc1, c-FOS, MMP-9, and CTSK, which suggested that OG inhibited osteoclast formation and differentiation in SAMP6 mice (Figure 3(a)).

The F-actin ring is recognized as the most remarkable character of osteoclasts during osteoclastogenesis and is indispensable for bone resorption. As seen in Figure 3(b), an apparent F-actin ring was observed in osteoclasts. However, the treatment with OG markedly decreased the amount and area of the F-actin ring. Thus, OG could suppress the formation of F-actin ring in osteoclast.

Osteoclastic bone resorption gives rise to deterioration of the bone matrix and subsequent release of Ca^2+^ and CTX-1 from the bone matrix. As shown in Figures 3(c)–3(e), the content of Ca^2+^ and CTX-1 in the medium was elevated after osteoclasts were incubated on the bone slices. OG decreased the content of Ca^2+^ and CTX-1 in the coculture medium of osteoclasts and the bone slice, suggesting that OG suppressed bone resorption. In addition, OG treatment markedly decreased the expression of NFATc1, c-FOS, CTSK, and MMP-9 in osteoclasts (Figure 3(f)). Taken together, these results indicate that OG suppresses bone resorption in osteoclasts.

### 3.4. OG Mitigates Oxidative Stress of Bone Tissue in SAMP6 Mice and Osteoclast Derived from RAW264.7 Cells

**Figure 4:**
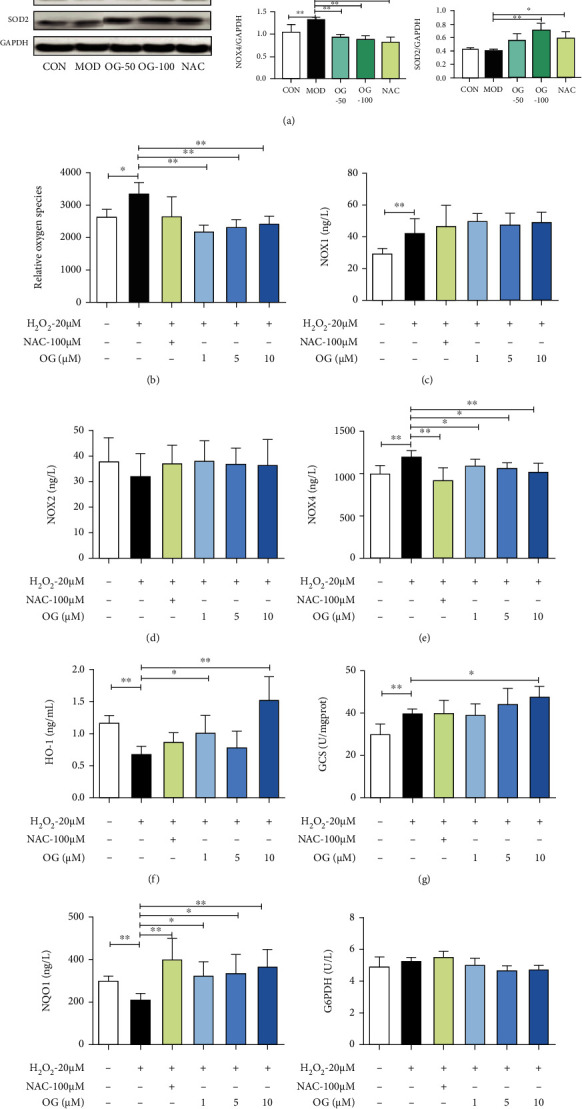
OG mitigates oxidative stress of bone tissue of SAMP6 mice and osteoclast derived from RAW264.7 cells. (a) Representative Western blot analysis and the quantification of NOX1, NOX2, NOX4, and SOD2 expression in bone tissue of mice. (b) Fluorometric quantification of ROS production was assayed using a multifunctional microplate reader; (c–i) the levels of NOX1, NOX2, NOX4, HO-1, GCS, NQO1, and G6PD were detected by ELISA kits. Images presented are representative of ≥3 sections for each group or independent experiments, and each point represents mean ± SD. ^∗^*P* < 0.05, ^∗∗^*P* < 0.01 vs. the indicated group.

To assess the effects of OG on oxidative stress in SAMP6 mice, related protein expression was detected. As indicated in Figure 4(a), the levels of SOD2 were markedly decreased in SAMP6 mice, and OG administration obviously elevated the expression of SOD2, and the expression of NOX2 and NOX4 was markedly increased in SAMP6 mice, while OG treatment dose-dependently decreased the levels of NOX2 and NOX4, but displayed no effect on the level of NOX1. Collectively, these data suggest that OG reduced oxidative stress of bone tissue in SAMP6 mice.

To further demonstrate the effects of OG on oxidative stress in osteoclasts, the levels of ROS, NOX1, NOX2, and NOX4 were examined in osteoclasts. As shown in Figures 4(b)–4(e), the DCF fluorescence intensity and the level of NOX4 in osteoclasts were increased in the treatment of RANKL and H_2_O_2_ but significantly decreased in the treatment of OG in a dose-dependent manner, demonstrating that OG retarded the oxidative stress in osteoclasts.

Moreover, the effects of OG on the antioxidant enzymes GCS, G6PDH, HO-1, and NQO-1 were determined. The results displayed that the levels of HO-1 and NQO1 were obviously decreased in H_2_O_2_-induced cells, but markedly overexpressed in OG treatment. In addition, the expression of GCS was obviously increased in H_2_O_2_-induced cells, and OG treatment elevated the activity of GCS, but exerted no effect on the level of G6PDH (Figures 4(f)–4(i)). All these founding further indicated that OG attenuated oxidative stress of H_2_O_2_-stimulated osteoclasts.

### 3.5. OG Upregulates Nrf2/Keap1 Pathway in Bone Tissue of SAMP6 Mice and Osteoclast Derived from RAW264.7 Cells

**Figure 5:**
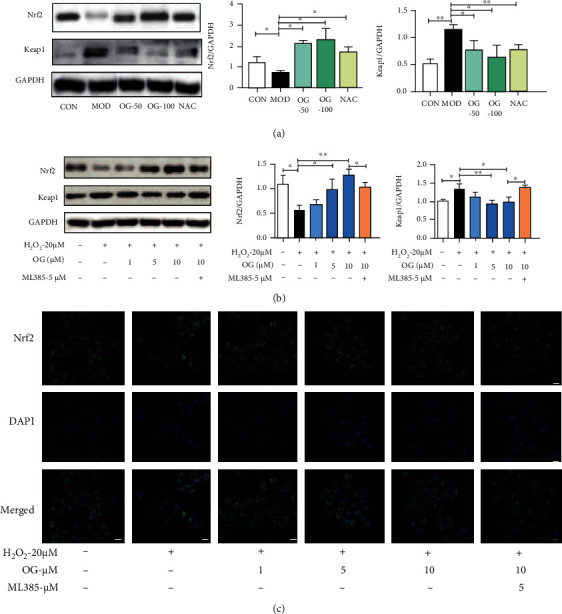
OG upregulates Nrf2/Keap1 pathway in bone tissue of SAMP6 mice and osteoclast derived from RAW264.7 cells. (a, b) Representative Western blot analysis and the quantification of Nrf2 and Keap1 expression in bone tissue of mice and osteoclast; (c) immunofluorescence analysis for nucleus translocation of Nrf2. Scale bars: 10 *μ*m. Each point represents mean ± SD (*n* = 3). ^∗^*P* < 0.05, ^∗∗^*P* < 0.01 vs. the indicated group.

To further demonstrate the underlying mechanisms of OG attenuating oxidative stress in osteoclastogenesis, we examined the protein expression levels of Nrf2 and Keap1 in bone tissue of SAMP6 mice and osteoclast derived from RAW264.7 and further determined nuclear translocation of Nrf2 in osteoclast. As shown in Figure 5(a), the results indicated that the expression of Nrf2 was increased but Keap1 decreased in SAMP6 mice under the treatment of OG. Meanwhile, as shown in Figure 5(b), RANKL and H_2_O_2_ treatment suppressed Nrf2 protein level and increased Keap1 expression in osteoclasts which were significantly reversed by the treatment of OG. Consistently, the immunofluorescence analysis also displayed a greater amount of Nrf2 protein localization in the nuclei after the treatment of OG (Figure 5(c)), which further validated the OG-mediated upregulation of Nrf2 activity. Furthermore, as an inhibitor of Nrf2 pathway, ML385 counteracted the activation and nucleus translocation of Nrf2 induced by OG (Figure 5). Taken together, our data showed that OG decreased oxidative stress of H_2_O_2_-induced osteoclast through upregulating the Nrf2 pathway.

### 3.6. OG Attenuates Autophagy in Osteoclast Derived from RAW264.7 Cells

**Figure 6:**
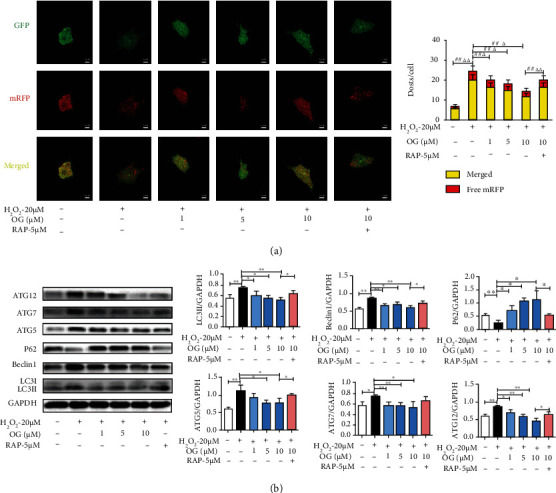
OG modulates the autophagy in osteoclast derived from RAW264.7 cells. (a) Representative images and quantitative analysis for yellow or red puncta per cell in osteoclast. Scale bars: 5 *μ*m. (b) Representative Western blot analysis and the quantification of LC3, Beclin1, p62, ATG5, ATG7, and ATG12 expression in osteoclast. The data are expressed as mean ± SD (*n* = 3). ^∗^*P* < 0.05, ^∗∗^*P* < 0.01, ^##^*P* < 0.01, *^Δ^P* < 0.05, *^ΔΔ^P* < 0.01 vs. the indicated group. ^##^ denotes the yellow puncta groups. *^Δ^* denotes the red puncta groups.

Autophagic flux was determined in osteoclast. The assay aimed to detect progression from autophagosome to autolysosome according to the pH sensitivity differences displayed by GFP and RFP between the acidic autolysosome and the neutral autophagosome. A phenomenon can be observed that the GFP moiety degrades while mRFP-LC3 maintains the puncta after the forming of autolysosome from the fusion of autophagosome and lysosome [24]. As shown in Figure 6(a), after transfection with Ad-mRFP-GFP-LC3, we found the successful introduction of this adenovirus showing both red and green fluorescent proteins. Apart from the accumulation of LC3, there were more red puncta in H_2_O_2_-induced osteoclast, which further demonstrated the induction of autolysosome formation, suggesting that H_2_O_2_ mediated autophagy flux in osteoclasts. Notably, OG treatment inhibited autophagy flux.

Moreover, Figure 6(b) shows LC3II, Beclin1, Atg5, Atg7, and Atg12 expression decreased and p62 expression increased in osteoclast of OG treatment group compared with the H_2_O_2_ group. Meanwhile, in order to reveal the relationship between autophagy and OG, rapamycin, a specific activator of autophagy, was used to activate autophagy. As shown in Figure 6(b), RAP significantly promoted the expression of Atg5, Atg7, Atg12, Beclin1, and LC3II protein and then decreased p62 expression in osteoclasts induced with H_2_O_2_. Taken together, these results showed that OG inhibited autophagy during osteoclastogenesis induced with a combination of H_2_O_2_ and RANKL.

### 3.7. OG Suppresses Autophagy by Activating mTOR Signaling in Bone Tissue of SAMP6 Mice and Osteoclast Derived from RAW264.7 Cells

**Figure 7:**
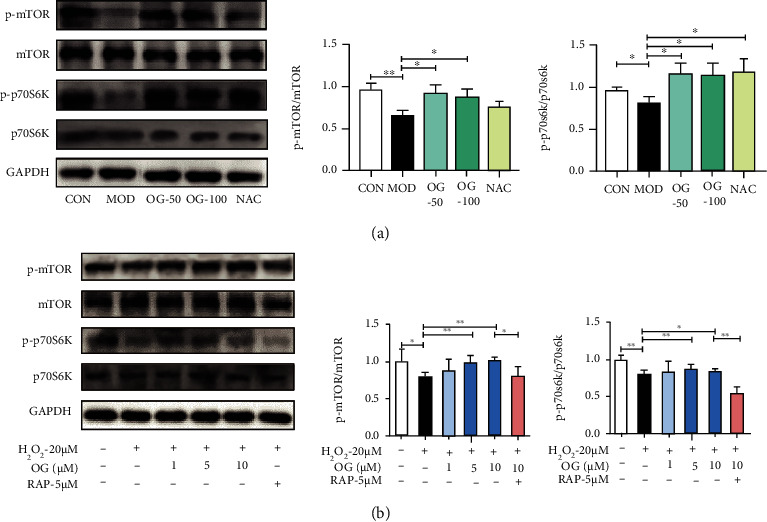
OG suppresses autophagy by activating mTOR signaling in bone tissue of SAMP6 mice and osteoclast derived from RAW264.7 cells. (a) Representative Western blot images and the quantification of p-mTOR, mTOR, p-p70S6K, and p70S6K expression in bone tissue of SAMP6 mice. (b) Representative Western blot images and the quantification of p-mTOR, mTOR, p-p70S6K, and p70S6K expression in the osteoclast with the treatment of OG or RAP incubation for 48 h. Each point represents mean ± SD (*n* = 3). ^∗^*P* < 0.05, ^∗∗^*P* < 0.01 vs. the indicated group.

Since mTOR was a vital regulator of autophagy, we examined whether mTOR signaling was involved in the OG effects of suppressing autophagy. Phosphorylation of mTOR and p70S6K was evaluated in the bone tissue. Compared with the control group, the model group showed decreased phospho-mTOR and phospho-p70S6K expression (Figure 7(a)). However, OG treatment increased the levels of phospho-mTOR and phospho-p70S6K in bone tissues of mice (*P* < 0.05).

To explore whether OG regulated mTOR signaling in osteoclasts, the expression of phospho-mTOR, total mTOR, phospho-p70S6K, and total p70S6K was analyzed by Western blot. After 48 h of OG treatment and subsequent 4 h of H_2_O_2_ treatment, the phosphorylation level of mTOR showed a significant decrease in response to RANKL and H_2_O_2_ exposure (Figure 7(b), *P* < 0.01). However, the effect was effectively recovered by OG at doses of 5 *μ*M and 10 *μ*M (*P* < 0.01). We also examined the activation of downstream p70S6K of mTOR signaling. OG treatment resulted in a steady increase in the phosphorylation level of p70S6K. Meanwhile, as shown in Figure 7(b), RAP significantly inhibited the phosphorylation of mTOR and p70S6K induced by RANKL and H_2_O_2_ in osteoclasts. These data suggested that OG might affect autophagy in RANKL and H_2_O_2_-induced osteoclasts through the mTOR signaling pathway.

### 3.8. The Interplay of Nrf2/Keap1 and mTOR Signaling Pathway in Osteoclast Derived from RAW264.7 Cells Is Dependently Regulated by OG

**Figure 8:**
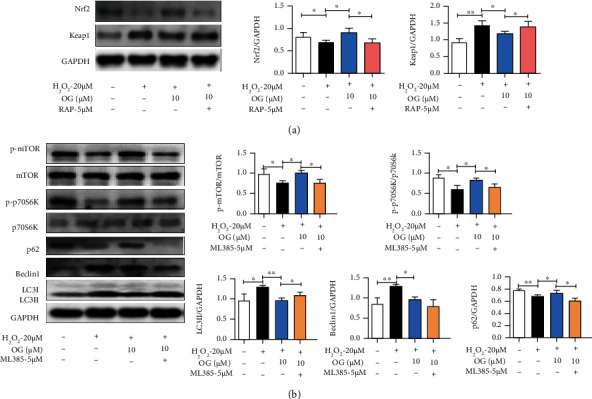
OG dependently regulates Nrf2/Keap1 signaling pathway and autophagy in osteoclast derived from RAW264.7 cells. (a) Representative Western blot images and the quantification of Nrf2 and Keap1 expression in osteoclast with the treatment of OG or RAP incubation for 48 h. (b) Representative Western blot images and the quantification of p-mTOR, mTOR, p-p70S6K, p70S6K, LC3II, Beclin1, and p62 expression in the osteoclast with the treatment of OG or ML385 incubation for 48 h. Each point represents mean ± SD (*n* = 3). ^∗^*P* < 0.05, ^∗∗^*P* < 0.01 vs. the indicated group.

To further clarify the role of autophagy in the OG-mediated Nrf2/Keap1 signaling pathway, we stimulated Nrf2 and Keap1 expression using an inhibitor of mTOR (RAP). Western blot analysis showed that the combination treatment with RAP obviously blocked the OG-induced activation on Nrf2 and suppression on Keap1 in osteoclast derived from RAW264.7 cells (Figure 8(a), *P* < 0.05).

On the other hand, to confirm the role of Nrf2/Keap1 signaling pathway in the OG-mediated autophagy mechanism, the inhibitor of Nrf2 (ML385) was used. Notably, the RANKL and H_2_O_2_-induced phosphorylation of mTOR and p70S6K and expression of LC3, Beclin1, and p62 were reversed by OG supplementation, but it was abrogated by ML385 (Figure 8(b), *P* < 0.05). These data verified that the suppression of autophagy triggered by OG was interdependent with the activation of Nrf2/Keap1 signaling pathway.

## 4. Discussion

As a consequence of bone-related aging diseases, SOP is one of the most common types of osteoporosis, which results in an increased risk to fragility fracture among the elderly. As the age grows, the level of ROS is increased and then leads to the enhancement of osteoclastic activity and bone destruction [25, 26]. The current study demonstrated that OG suppressed bone loss in SAMP6 mice and osteoclastogenesis from macrophagic RAW264.7 cells under the induction of RANKL and H_2_O_2_ through attenuating oxidative stress and autophagy via activating the Nrf2/Keap1 and mTOR signaling pathway.

The SAMP6 mice have been commonly used as the spontaneous experimental model for senile osteoporosis, which shows a bone phenotype consistent with that of human senile osteoporosis, including reduced trabecular bone volume, decreased BMD, and reduced bone strength [27]. In senile osteoporosis, bone remodeling is diminished with increasing age, subsequently resulting in the negative balance between bone resorption and formation [9]. As it is reported that bone loss in SAMP6 mice results from decreased bone formation and increased bone marrow adiposity [28], the changes in bone resorption remain unclear. The present study found that osteoclastic bone resorption was increased in SAMP6 mice, and treatment with OG improved the bone microarchitecture and increased the BMD and especially decreased the number of TRAP-positive osteoclast in SAMP6 mice, also suppressing the osteoclastogenesis of RAW264.7 cells induced by a combination of RANKL and oxidative stress, exhibiting that OG counteracted accelerated bone loss through inhibiting osteoclastic bone resorption in SAMP6 mice.

The senescence is associated with increased oxidative stress and elevated levels of ROS. Emerging research has displayed that oxidative stress is involved in the regulation of osteoclastogenesis [29, 30]. In order to understand the oxidative stress state in the skeleton of SAMP6 mice, we analyzed the oxidative and antioxidative enzymes in bone tissue of SAMP6 and found that the activities of oxidases NOX2 and NOX4, which could promote osteoclastogenesis in osteoclast or mice [31, 32], were significantly increased, and levels of SOD were significantly decreased, suggesting a higher level of oxidative stress in the skeleton of SAMP6 mice. Treatment with OG attenuated the oxidative stress in the bone of SAMP6 mice. Moreover, OG declined the levels of ROS and NOX4 in osteoclast derived from RAW264.7 cells under oxidative stress, also suggesting that OG alleviated oxidative stress and inhibited osteoclastogenesis.

The accumulating evidence showed that Nrf2, as ROS negative regulators, is involved in the regulation of oxidative stress in levels of cell and tissue [33]. Usually, Keap1 negatively regulates Nrf2-dependent expression of antioxidative enzymes, such as HO-1, GCS, NQO1, and G6PD, by suppressing nuclear translocation of Nrf2, and these enzymes counteract generations of ROS and attenuate oxidative stress [34]. During the osteoclastogenesis induced by RANKL, Keap1 dissociates from Nrf2, causing the latter to escape and enter the nuclear, where it binds to the promoter region of the antioxidative response element (ARE) to promote the expression of antioxidant enzymes [35]. The present study found that the expression of Nrf2 was reduced and the expression of Keap1 was enhanced in bone tissue of SAMP6 mice, showing a decreased capacity of defensing oxidative stress. Treatment with OG reversed the alteration of Nrf2 and Keap1 in the bone of SAMP6 mice and osteoclast and also elevated the activities of cytoprotective enzymes in oxidative stress of osteoclast derived from RAW264.7, indicating that OG can potentially scavenge ROS via activating the Nrf2 pathway.

Aging is a major risk factor for osteoporosis, and this event is directly associated with autophagy. Accumulating evidence shows that autophagy might be a nonnegligible potential target for regulating osteoclastic differentiation [36, 37]. Particular conditions, such as inflammation and oxidative stress, could stimulate autophagy in preosteoclasts and influence osteoclastogenesis, leading to pathological changes [38]. Fortunately, autophagy can be pharmacologically regulated by certain agents. For example, RAP, an inhibitor of mTOR which negatively regulates autophagy, can induce autophagy [39]. The present study found that autophagy was increased in the osteoclast of bone tissue in SAMP6 and osteoclast induced with oxidative stress. These findings were consistent with the previous studies, showing that autophagy is activated during osteoclastogenesis [37, 40]. Treatment with OG decreased autophagy of osteoclast derived from RAW264.7 cells, revealing that OG was involved in the regulation of autophagy, and then reduced the function of osteoclast during the process of aging.

As a redox-sensitive factor, mTOR was a negative regulator of autophagy. Several studies have reported that mTOR plays a critical role in mediating osteoclastogenesis and autophagy [41, 42]. Furthermore, the mTOR signaling was constitutively inactivated in osteoclast induced by oxidative stress. In line with these reports, our results showed aging-induced decreases in levels of p-mTOR and p-p70S6K in bone tissue of SAMP6 mice and the oxidative stress-exposed osteoclasts. The phosphorylation of mTOR and p70S6K was upregulated by OG treatment in bone tissue of SAMP6 mice and the oxidative stress-exposed osteoclasts, demonstrating that OG inhibited autophagy of osteoclast via modulating mTOR signaling pathway.

The increasing evidence demonstrates that Nrf2 and mTOR signaling pathways are interconnected to modulate oxidative stress and autophagy [43]. Nrf2 positively regulates mTOR, and the downregulation of the Nrf2 expression results in the suppression of mTOR in osteoclasts. In line with these reports, our results showed that RAP negatively regulated the activation of OG on Nrf2/Keap1 signaling, and ML385 reversed the suppression of OG on autophagy. Collectively, our data highlight the suppression of autophagy and activation of Nrf2/Keap1 signaling are interlinked during OG-mediated antiosteoclastogenesis.

Accumulating evidence showed that p62, a multifunctional scaffold protein, takes part in the regulation of osteoclastogenesis. Ubiquitination of TNF receptor-associated factor 6 (TRAF6) triggers RANK-induced osteoclastogenesis through NF-*κ*B pathway, and the p62-TRAF6 complex is crucial for the optimal and sustained activation of NF-*κ*B [44]. On the other hand, when p62 mediates the binding of deubiquitinating enzyme CYLD and TRAF6, CYLD could negatively regulate RANK signaling by suppressing TRAF6 ubiquitination and activation of downstream signaling events [45]. P62 mutations in ubiquitin-binding domain have been reported to be closely related to Paget's disease which is characterized by the formation of giant osteoclasts with a remarkable increase in bone resorption [46]. In addition, p62 is also involved in Keap1/Nrf2 pathway which is manifested that p62 phosphorylation activates the expression of cytoprotective Nrf2 targets [47]. Our results displayed OG upregulated the expression of p62 accompanied by the autophagy inhibition in osteoclasts, thus further studies on the p62-mediated integrated regulation of autophagy, Nrf2 and NF-*κ*B pathway in osteoclasts may be helpful to deeply demonstrate the effect of OG and even provide new therapeutic targets for the osteoclast-related disease.

## 5. Conclusions

**Figure 9:**
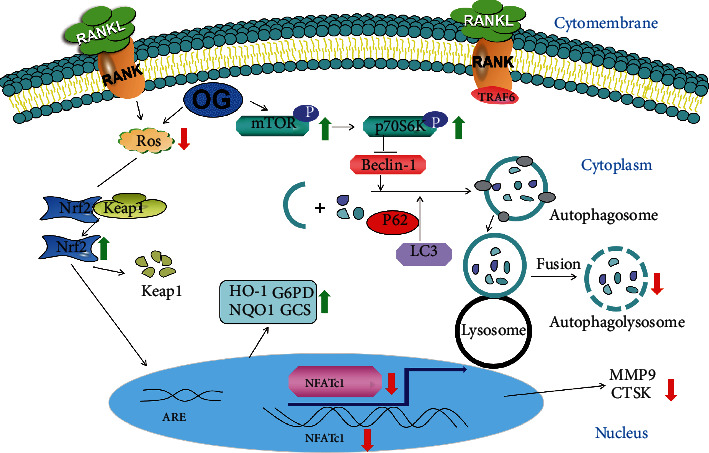
A schematic diagram illustrating the potential mechanism of orcinol glucoside (OG)'s effects in a mouse model of senile osteoporosis. OG inhibited osteoclastogenesis by scavenging ROS, subsequently elevating the expression of antioxidant enzymes via activating the Nrf2/Keap1 pathway and inhibiting autophagy by activating the mTOR pathway. Thus, OG is a promising candidate drug for the treatment of bone disorders related with aging and oxidative stress, especially senile osteoporosis in the future.

In summary, our study both in vivo and in vitro suggested that OG could serve as an inhibitor of osteoclastogenesis by scavenging ROS, subsequently elevating the expression of antioxidant enzymes via activating the Nrf2/Keap1 pathway and suppressing autophagy by activating the mTOR pathway (Figure 9). The current study provides convincing evidence that OG may be a promising candidate drug for the treatment of bone disorders related with aging and oxidative stress, especially senile osteoporosis in the future.

## Data Availability

The data that support the findings of this study are available from the corresponding author upon reasonable request.
